# Harnessing the Power of Antimicrobial Peptides: From Mechanisms to Delivery Optimization for Topical Infections

**DOI:** 10.3390/antibiotics14040379

**Published:** 2025-04-04

**Authors:** Songhita Mukhopadhyay, Souha H. Youssef, Yunmei Song, Usha Y. Nayak, Sanjay Garg

**Affiliations:** 1 Centre for Pharmaceutical Innovation, Clinical and Health Sciences, University of South Australia, Adelaide, SA 5000, Australia; songhita.mukhopadhyay@mymail.unisa.edu.au (S.M.); souha.youssef@unisa.edu.au (S.H.Y.); may.song@unisa.edu.au (Y.S.); 2 Department of Pharmaceutics, Manipal College of Pharmaceutical Sciences, Manipal Academy of Higher Education, Manipal 576104, Karnataka, India; usha.nayak@manipal.edu

**Keywords:** antimicrobial resistance, antimicrobial peptide, proteolytic degradation, self-assembly, skin microbiome, topical infection

## Abstract

Antimicrobial peptides (AMPs) have emerged as promising agents for treating topical infections due to their enhanced biocompatibility and resistance to systemic degradation. AMPs possess host immunomodulatory effects and disintegrate bacterial cell membranes, a mechanism less prone to microbial resistance compared to conventional antibiotics, making AMPs potential candidates for antimicrobial delivery. The review discusses the challenges posed by antimicrobial resistance (AMR) and explores the mechanisms by which bacteria develop resistance to AMPs. The authors provide a detailed analysis of the mechanisms of action of AMPs, their limitations, and strategies to improve their efficacy. Conventional AMP delivery systems, including polymeric, synthetic, and lipid-based nanoparticles and cubosomes, face challenges of microbial resistance mechanisms via efflux pump systems, bacterial cell membrane modifications, and protease enzyme release. This review explores strategies to optimize these delivery systems. Furthermore, market statistics and the growing interest in peptide antibiotics have been explored in this review. The authors provide future research directions, such as exploring gene-targeting approaches to combat emerging bacterial resistance against AMPs, and emphasize considering the conformational stability of peptides, the skin microbiome’s nature at the infection site, and proteolytic stability for developing efficient AMP delivery systems for topical infections.

## 1. Introduction

Antimicrobial resistance (AMR) has become a primary focus for current pharmaceutical researchers due to its growing niche of innovative drug delivery technologies. The increased resistance (up to 73% of infectious diseases), specifically against the ESKAPE pathogens (*Enterococcus faecium*, *Staphylococcus aureus*, *Klebsiella pneumoniae*, *Acinetobacter baumannii*, *Pseudomonas aeruginosa*, and *Enterobacter species*), has become a serious global threat [1,2]. Addressing AMR is essential for both systemic and topical infections.

Being the fourth most common cause of human disease, topical infections are often underrated [3], starting from common skin ailments that can be bacterial, viral, or fungal to serious chronic skin infections, namely cellulitis and soft tissue infections [4]. Bacterial infections represent most of the common skin infections [4]. Several topical antibacterial agents, such as mupirocin, bacitracin, fusidic acid, polymyxin B, and neomycin, are currently on the market indicated for most staphylococcal skin infections [4,5]. However, growing resistance via different mechanisms, such as horizontal gene transfer and encoding specific metalloproteins, avoids the binding of these antibacterial agents [4].

Antimicrobial peptides (AMPs), specifically short peptides (5–15 amino acids), have shown promise in combating bacterial resistance [4]. These naturally occurring AMPs (host defense peptides) possess a cationic nature and exert a strong electrostatic interaction with the negatively charged bacterial surface, leading to disruption of the bacterial cell membrane [6]. Various models of AMP targeting, such as the barrel stave, carpet model, and toroidal pore model, highlight the bactericidal activity of these peptides [7]. A more detailed classification of AMPs has been reviewed by Bin Hafeez et al. [8]. Briefly, this process begins with electrostatic interactions between the cationic charge of AMPs and the negatively charged bacterial surface, such as anionic phospholipids in the cell membrane and lipopolysaccharide (LPS) in Gram-negative bacteria or teichoic acid in Gram-positive bacteria [6,7]. Following this attraction and attachment, AMPs disrupt the integrity of the bacterial cell membrane through several proposed models, namely the barrel-stave model where AMPs insert themselves across the lipid bilayer, the carpet model where, unlike barrel stave, AMPs do not insert themselves across the membrane but instead cover the membrane surface like a carpet. In the Toroidal pore model, AMPs insert into the lipid bilayer and induce the lipid monolayers to bend. This bending creates a pore-like structure [6,7]. These mechanisms lead to permeabilization or disintegration of the microbial cell membrane, ultimately causing cell death.

Several technologies are being explored to enhance AMP efficacy and stability. Structurally nanoengineered antimicrobial peptide polymers (SNAPPs) utilize the bactericidal activity of naturally occurring AMPs [7]. Currently, several AMPs are undergoing clinical trials [9], but the challenge of improving their stability is the area where pharmaceutical researchers are mostly invested [4].

A robust line of peptide antibiotic products is anticipated to contribute to the growth of the peptide-antibiotic market [10,11,12]. As per the current market (Table 1) of peptide antibiotics, the skin infection segment accounted for the largest revenue share of 30.3% in 2021 and is expected to continue leading the market throughout the forecast period (2022–2030) [10]. Joint ventures between companies like Boehringer Ingelheim and BioMérieux are expected to drive the development of the next generation of antibiotics. The market share for the skin infection segments of the AMPs is likely to dominate in the coming years [13].

Despite the potential of AMPs, the cost efficiency (production) and resistance to such peptides have been a limitation. Thus, utilizing recombinant engineering methods from prokaryotes can help reduce these costs. Resistance to such AMPs has also been addressed by synthesizing derivative peptides. However, efficient susceptibility testing against most of the common pathogens like methicillin-resistant *Staphylococcus aureus* (MRSA) should pave the path for AMP production and reduce the risk of resistance development against such AMPs [4,13].

Several reviews have been reported for emerging treatments for topical infections utilizing AMPs [4,6,14,15]. Specifically, metallic nanoparticles [16,17], carbon-based nanomaterials [16], and hydrogels [6,16]. The use of metallic nanoparticles to enhance AMP properties, namely stability, toxicity, half-life, and release profile, has been specifically discussed in the literature [17]. The potential of AMPs to treat multi-drug resistant and biofilm-forming bacteria and fungi in wound infections via polymers, scaffolds, films, and nanoparticles has been discussed in the literature [18]. However, such existing literature does not discuss specific aspects like the effects of such delivery systems on the skin microbiome [16], limited scope of other delivery systems [17], a brief discussion on bacterial resistance mechanisms to AMPs, and a limited explanation of the advantages and disadvantages of each delivery strategy [18]. Additionally, the existing literature [6] does not mention any marketed formulations for topical AMP applications. A key focus area on certain aspects of AMP delivery, which includes maintaining the stability within the formulation and increasing the contact time of these emerging therapeutics, has not been addressed.

This review addresses the gaps of such existing review articles; namely, it has a focus on the skin microbiome, which is important while designing peptide delivery systems. It highlights factors such as pH and its influence on the local concentration of AMPs. Secondly, there is an emphasis on the need to maintain the peptide conformation stability during formulation development, which has received less attention in other review articles [6,16,17,18]. Understanding the commercial implications of AMP therapeutics has been explored in this review, as mentioned in market statistics (Table 1). While all existing literature [6,16,17,18] acknowledges the limitations of current AMP delivery therapies, this review provides a concise summary of these limitations, encompassing microbial resistance mechanisms, short residence time, bioadhesivity issues, and cytotoxic concerns. This reinforces the need for optimization strategies to improve the current AMP-derived formulations for topical infections.

**Table 1:** Marketed topical AMP formulations.

Marketed Product	Type of Product	Company	Target Disease	Reference
Cubicin RF	Lipopeptide	Merck & Co., Inc. (Rahway, NJ, USA)	Skin infections	[19]
Daptomycin (cubicin) IV 4 mg/kg	Cyclic Lipopeptide	AuroMedics Pharma LLC (East Windsor, NJ, USA)	Skin infections	[20]
Polymyxin B vials	Polypeptide antibiotics	Xellia (Copenhagen, Denmark)	Acute urinary, meningeal or blood stream infections	[21,22]
Vancocin (vancomycin hydrochloride (1–2%)	Glycopeptides		Septicemia	[23]
Dalvance/allergan (dalbavancin 500 mg/vial)	Second-generation lipoglycopeptide antibiotic	Melinta Therapeutics (Parsippany-Troy Hills, NJ, USA) FDA approval May 2014	Acute skin structure infections	[24]
Telavancin	Semisynthetic peptide derivative	Theravance Biopharma (South San Francisco, CA, USA)	Serious bacterial skin infections	[25,26]
Orbactiv (oritavancin)	Semisynthetic lipoglycopeptide	Melinta Therapeutics	Acute skin structure infections	[27]
Omiganan pentahydrochloride	Synthetic analog of human defensin		Atopic dermatitis	[28,29]

## 2. Resistance to Antimicrobial Peptides

The lipid bilayer of the bacterial cell membrane provides an efficient line of defense against various antimicrobials. This threat of antimicrobial resistance has been augmented by several other factors like inappropriate use of antibiotics, cross-contamination in hospital setup, lack of drug efficacy, and evolving mutagenesis within the ESKAPE pathogens [1].

**Figure 1:**
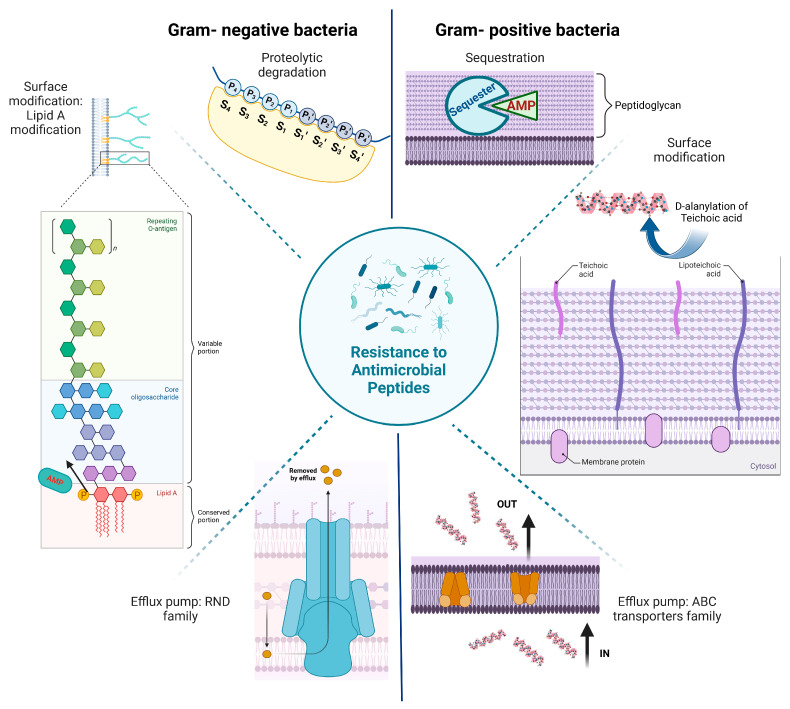
Mechanisms of resistance to antimicrobial peptides “Created with BioRender.com”.

The rapid evolution of antimicrobial-resistant mutants renders the utilization of AMPs as a potential antimicrobial agent attractive. The unique mechanism of bacterial membrane perturbation and disintegration by AMPs makes it difficult for bacteria to develop resistance [6,7]. However, resistance to AMPs has also been triggered in Gram-positive and Gram-negative bacteria via several mechanisms like proteolytic degradation, efflux pump systems, and cell surface alterations (Figure 1) [30].

From a broader perspective, bacteria show resistance to AMPs in both passive and adaptive manner [31]. A passive mechanism arises due to the presence of an inherent positively charged moiety known as Lipid A outside the bacterial cell membrane, which reduces the interaction of the cationic peptides with the membrane surface [31]. Several passive mechanisms of resistance, such as ATP-binding cassette (ABC) transporters of Gram positives like *Staphylococcus aureus* and *BceAB* type two-component ABC transporters, are active against a broader range of AMPs [30]. In the context of superficial skin bacterial infections, the *VraFG* ABC transporter of *Staphylococcus aureus* (causative organism of skin infections like impetigo and most skin and soft tissue infections) elicits resistance to a wide range of AMPs [30]. Bacteriolytic proteins or proteases, which play an important role in AMP resistance, are secreted by a wide range of *Group A Streptococcus*, *Enterococcus*, metalloproteases from *Staphylococcus aureus*, and Gram-negatives such as *Pseudomonas aeruginosa.* Extracellular surface modifications against a broad range of AMPs, including lantibiotics, polymyxins, and colistin, have been reviewed [31,32,33].

The second type of resistance mechanism that is common against AMPs is the adaptive or inducible mechanism of resistance, where a modification of the extracellular bacterial surface at a molecular level occurs [34]. The D-ala-D-ala residue of the peptidoglycan is substituted with D-lactate, thus reducing the interaction of some antibiotics such as vancomycin with the bacterial cell membrane [35]. Detailed resistance mechanisms against AMPs are out of the scope of this paper. Comprehensive papers are available for readers with a keen interest [30,31]

## 3. Skin Microbiome

The skin, as the body’s first line of defense against microbes, hosts a diverse microbiome, including commensals, pathogens, residents, transients, mutualistic microbes, and opportunistic pathogens [36]. This classification served as the basis for the “Human Microbiome Project”, which utilized RNA gene sequencing techniques from healthy volunteers to generate critical data on the nature of the healthy skin microbiome. This dataset is used for the detection of pathogens that are activated or unmasked during specific disease conditions [36]. AMPs play an integral role due to the correlation between AMP levels and the severity of bacterial infections. The reduction in naturally occurring AMPs in skin infections like atopic dermatitis (AD) contributes to unhindered bacterial infections, making the detection and study of such patterns crucial for disease management [36].

**Figure 2:**
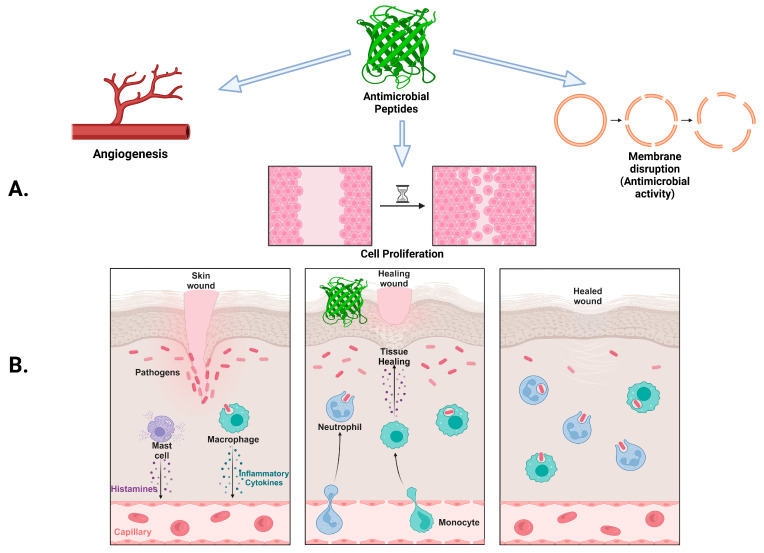
Function of antimicrobial peptides (AMPs): (**A**) AMPs in wound healing stages; (**B**) AMPs generating an immunogenic response to skin injury “Created with BioRender.com”.

The coordinated and timely completion of all four biological processes (hemostasis, inflammation, proliferation, and remodeling) [37] is essential for successful wound healing (Figure 2). Direct antimicrobial activity by bacterial membrane disruption, enhancement of cell migration and proliferation, induction of neovascularization, and functioning as potent immunomodulators encompasses the powerful and complicated mechanism of action of AMPs (Figure 2) [38].

## 4. Significance of pH in AMP Delivery for Topical Infections

The topographical variation of a healthy skin microbiome is comprised of various endogenous factors like pH and localized concentration of microbial species affecting the selection of the appropriate AMP delivery system. Considerable research has been conducted on bacterial membrane interactions with peptides and their bactericidal effects [6,7,15]. Bacteria in the wound site express toxins and other protease enzymes, which leads to low metabolic stability of these therapeutic peptides [6,15]. Thus, effective transdermal AMP delivery remains a challenge. The knowledge of pH variations during wound healing is critical, as it could affect the extent of expression of microflora, leading to inflammatory diseases, including AD, impetigo, and diabetic foot ulcers. The cutaneous pH range of 5.5–6 changes upon infection [39], which in turn affects the whole series of tissue remodeling processes (comprising of cell migration and proliferation) [6,39]. It is evident that lower pH favors wound healing [40]. Thus, indicating that higher pH is an important marker in detecting skin infections in the preliminary stages [39,40].

## 5. Key Factors to Be Considered for Novel AMP Delivery

AMP activity depends on factors like safety, concentration, stability, and the pH of its surroundings [41,42,43]. Transdermal delivery is the most viable method for administering AMPs because it ensures a localized and higher concentration of peptides at the infection site [44]. However, emerging AMP delivery systems in wound healing (nanoparticles, cubosomes, and nanostructured lipid carriers) suffer from several limitations, such as limited bioadhesivity, low residence time, fibroblast cell toxicity leading to biocompatibility, biodegradability issues, and degradation of peptides [6]. The following section of emerging AMP delivery systems (nanoparticles, cubosomes, and nanostructured lipid carriers) discusses existing AMP delivery systems with an insight into their limitations.

## 6. Emerging AMP Delivery Systems

### 6.1. Nanoparticles

**Figure 3:**
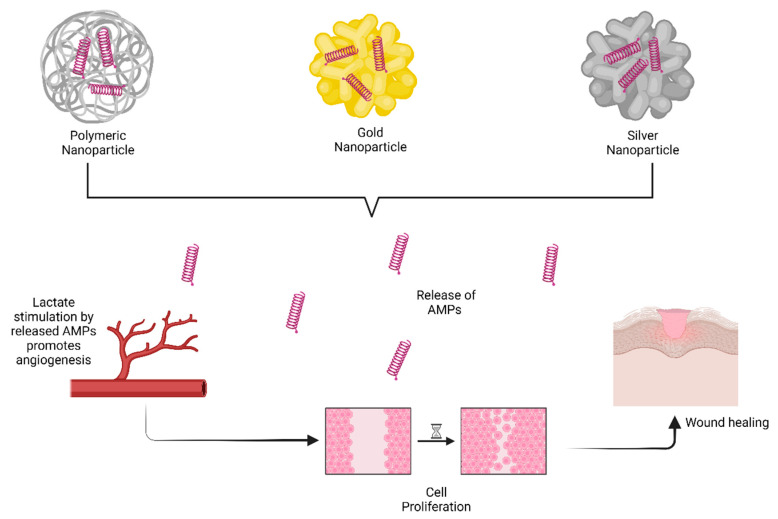
Nanoparticles in topical delivery of AMP “Created with BioRender.com”.

The advantage of higher encapsulation efficiency and improved pharmacokinetic profile has increased the pace of development of nanoparticles (NPs) in the field of drug delivery [6]. Several forms of NPs were developed from different sources, namely natural (chitosan-based [45,46]) and synthetic (PLGA [47] and gold nanoparticles [48]), which have been widely explored and investigated in the literature. NPs (polymeric, gold, and silver) in topicals promote the process of wound healing by targeting a factor, lactate, responsible for one of the important biological processes in wound healing, i.e., cell remodeling and regeneration [6,49]. The literature suggested that reactive oxygen species, such as lactate (an end product of anaerobic glucose metabolism), play a significant role in the underlying processes of wound healing (renewal and regeneration) [50]. Thus, significant levels of lactate can stimulate angiogenesis [6]. Sustained and improved release of AMPs via NPs as delivery mechanisms induces improved lactate stimulation, which further leads to cell proliferation and migration and wound healing (Figure 3). However, limited residence time is the major disadvantage of such AMP-based NPs, which can be detrimental to chronic wounds like diabetic foot ulcers [47].

Fibroblast cell cytotoxicity is induced by gold NPs, which can limit the normal wound-healing process [51]. Gold NPs [52] contain gold, which makes them unsuitable from the perspective of biocompatibility and biodegradability for such NPs [6]. Silver nanoparticles, on the other hand, tend to precipitate out free silver ions in the stratum corneum layer, making them less preferable than gold NPs. However, due to the aforementioned cell cytotoxicity, gold NPs are associated with issues as an AMP delivery system [6,53]. Additionally, augmented macrophage release triggered by the adsorption of gold NPs into wounds can generate inflammatory responses [54,55]. Limited bioadhesivity and minimal residence time are other drawbacks for such gold and silver nanoparticles [6].

### 6.2. Cubosomes

Cubosomes are amphipathic three-dimensional spatial arrangements with interwoven water channels consisting of folded lipid bilayers, thus serving the advantage of incorporating hydrophilic, hydrophobic, and amphiphilic molecules within their structure [56]. AMPs such as AP114 [57,58,59], human kininogen derivative DPK-60 [57,58], gramicidin A, melittin, alamethicin [60], and human cathelicidin LL-37 [61,62] have been incorporated in cubosomes. Being an important part of the innate immunity system of the skin and exhibiting wound-healing properties, LL-37 has been the preferred candidate for topical treatment of bacterial infections [63,64,65].

**Figure 4:**
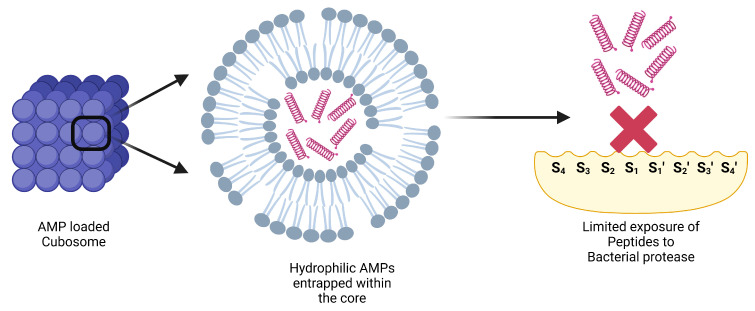
Cubosomes in topical delivery of AMP “Created with BioRender.com”.

LL-37-based cubosomes for wound infections have been explored for antimicrobial efficacy against *Staphylococcus aureus*, *Pseudomonas aeruginosa*, and *Escherichia coli* [66]. Three methods were used to prepare AMP-based cubosomes. Firstly, a pre-loading method involved developing a liquid crystalline gel incorporating LL-37, which was then dispersed into cubosomes. Secondly, the post-loading approach entailed adsorbing LL-37 onto pre-existing cubosomes. Lastly, the hydrotrope-loading technique involved using a spontaneous mixture of ethanol and glycerol monooleate for loading LL-37 [66]. The pre-loading method demonstrated the most promising results due to the hydrophilic-hydrophobic interactions between the water channels of the cubosomes and the entrapped peptide, thus limiting exposure of the peptides to bacterial elastases (Figure 4), unlike the loading technique, where peptides are exposed over the surface, hence making it prone to proteolytic degradation [66]. Nevertheless, this novel delivery system of cubosomes could not resolve the issue of limited exposure at the local site of infection. Furthermore, excipient selection during the cubosome production for topical peptide delivery requires special attention towards the wound microenvironment, as the chronic wound microenvironment can be highly sensitive towards certain excipients [6,66].

### 6.3. Nanostructured Lipid Carriers (NLCs)

**Figure 5:**
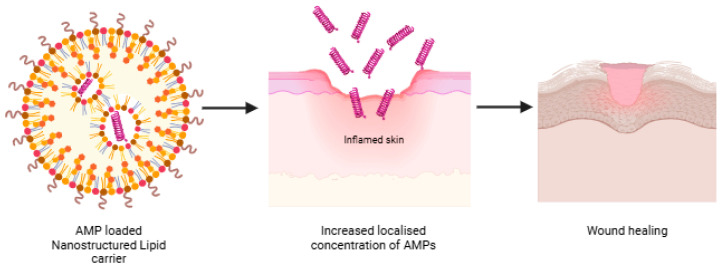
Nanostructured lipid carrier in topical delivery of AMP “Created with BioRender.com”.

High levels of AMPs are essential for combating chronic wound infections and fostering effective healing. With the advantage of increased encapsulation efficiency (up to 96%) and elevated levels of localized concentration of AMPs (Figure 5), LL-37-based NLCs have been investigated over wound models of mice [67]. A melt emulsification technique was used to prepare these particles over a size range of 270 nm, and significant bioactivity was observed after the encapsulation of peptides in the lipid-based system. However, such systems have major drawbacks of stability in aqueous systems and restricted stay following topical application [6]. Moreover, they trigger reactive oxygen species (ROS) production upon lipid-based nanoparticle degradation. Thus hindering the wound-healing process via oxidative stress [6,68,69].

## 7. Key Strategies to Improve AMP Delivery for Topical Infections

As mentioned earlier, bioadhesivity, residence time, biocompatibility, conformational stability, the effect of the microenvironment, and pH considerations are important factors to be considered for potential AMP delivery via topical mode. There have been ample studies attempting to balance these factors to optimize efficient formulation delivery of AMPs (Table 2 and Table 3).

### 7.1. Hydrogels

**Figure 6:**
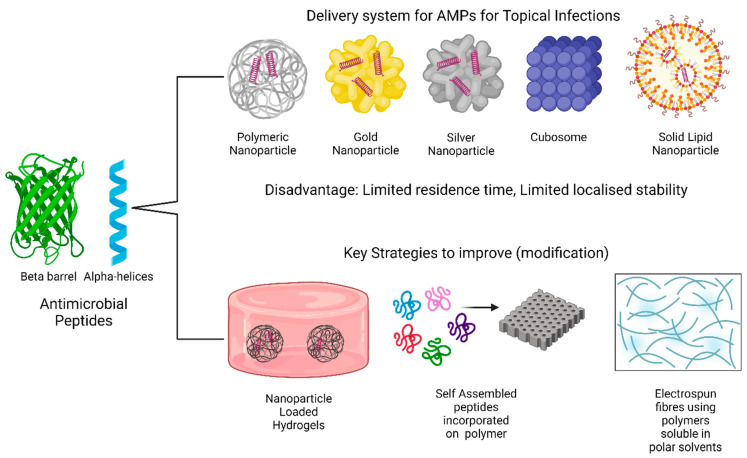
Key Strategies to improve AMP delivery for topical infections “Created with BioRender.com”.

Three-dimensional polymer networks with high water content, known as hydrogels, have shown promising proof-of-concept for the delivery of AMPs for effective wound healing applications (Figure 6) [6,70]. The improvement of such systems by aligning them towards being more skin microenvironment-friendly has been achieved via stimuli-responsive hydrogels [70]. AMPs susceptible to hydrolysis, oxidation, and light can be encapsulated in NPs and then embedded in hydrogels to protect against degradation. This approach extends the duration that AMP-loaded NPs remain at the infection site. This combination effect is more effective than bare nanoparticles [70]. Similar observations were noted using *Staphylococcus aureus* as a model pathogen where NP-stabilized liposomes were incorporated into a hydrogel, resulting in a sustained topical drug delivery system with no skin toxicity during seven-day treatment [71]. AMPs can be incorporated into hydrogels, including simple mixing within the polymer network, ionic interactions with the hydrogel material, and covalent conjugation to the polymer chains [6]. Stimuli-responsive hydrogels based on environmental cues such as pH, temperature, or the presence of specific enzymes allow for targeted and on-demand delivery of AMPs [6]. The release of AMPs from the hydrogels occurs through mechanisms including diffusion through the hydrogel matrix, swelling of the hydrogel, or chemical degradation of the hydrogel network, leading to the release of the entrapped AMPs [6]. The rate of AMP release can be tailored by adjusting the properties of the hydrogel, such as the degree of polymer cross-linking, the chemical structure of the monomers, and the intensity of external stimuli in the case of responsive hydrogels [6]. For instance, higher cross-linker concentrations generally lead to a decreased release rate [71].

The primary role of hydrogels is to serve as a delivery vehicle and a protective matrix for the AMPs [6]. It enhances the effectiveness of AMPs by providing higher AMP concentration at the target area [6]. In the case of nanoparticle-embedded hydrogels [71], the hydrogel first releases the AMPs through diffusion, which interacts with the bacteria, and the AMPs exert their antimicrobial action [71]. Hydrogels also contribute to prolonged contact time of the AMPs with the target area [6].

In summary, hydrogels facilitate AMP delivery by providing a protective environment, localizing the AMP action at the target site, thereby enhancing the AMPs’ inherent antimicrobial mechanism of action. They serve as a crucial tool to overcome the limitations of bare AMPs, such as susceptibility to degradation and short residence time.

### 7.2. Self-Assembling Peptides

The structural and compositional versatility of peptide nanomaterials establishes the idea for designing self-assembling peptides promising better conformational stability of AMPs (Figure 6) [72]. The effect of self-assembling peptides has shown a broader spectrum antimicrobial effect, including multidrug-resistant (MDR) ones. The mechanism of cell lysis via pore formation (barrel-stave model) of antimicrobial peptides, namely alamethicin, is more efficient via self-assembling [73]. The process of AMP delivery via self-assembling peptides involves two approaches. One approach involves conjugating AMPs to self-assembling peptide sequences, which further form nanostructures and act as carriers for the AMP [72]. Another is by assembling AMPs with other biomaterials to enhance their properties and delivery [73]. The concept of enzyme-instructed self-assembly (EISA), where enzymes trigger the self-assembly of peptide precursors, has also been explored [72]. Both the hydrophilic and hydrophobic properties of the amino acids within the self-assembling peptide sequence play a crucial role in the self-assembly process and the resulting nanostructure’s morphology [72].

However, the toxicity and limited enzymatic stability of such peptides are major concerns to be considered, which have been addressed via the incorporation of such self-assemblies into thermosensitive polymeric carriers [73].

### 7.3. Other Strategies to Improve AMP Delivery Systems

Regarding the formulation of AMPs, wafers have been an interesting approach that solves the limited contact time issue presented by cream and ointment formulations of some AMPs [6]. Initially, the flexibility of AMP needs to be considered, which is challenging for wafers. However, using a flexible linker, conjugation of AMPs over the surface of wafers has been shown to address this limitation [74].

Wound dressings and other skin-pertaining delivery systems, for instance, electrospun fibers for AMP delivery, offer better exude absorption, oxygen permeability, and enhanced cell proliferation. However, most of these systems have been focusing more on small molecules, whereas considerably less attention has been placed on AMPs. The reason is that the hydrophobicity of polymers like polycaprolactone (PCL), which is used in electrospinning, are incapable of solubilizing charged AMPs. However, by combining with other hydrophilic polymers, the possibility of incorporating AMPs can be improved [75]. For example, polyethylene oxide (PEO) or polyvinyl alcohol (PVA) can electrospin in polar solvents, which renders compatibility with AMPs. Thus, promoting dissolution and swelling of peptides (Figure 6) [75].

Multiple delivery strategies have been under investigation for improving the existing AMP delivery systems either by modifying the starting material or AMP, by modifying the formulation process (Table 2 and Table 3), and by completely establishing new delivery systems for AMPs such as mesoporous silica nanoparticles (MSNs), antimicrobial peptide conjugates (APCs), bacteria-absorbing sponges, layered nanoclays, and titanium nanoparticles (Table 3).

**Table 2:** Strategies to improve novel AMP delivery for topical infections.

AMP	Limitation	Strategies	Results	Reference
LL-37	Proteolytic degradation	New AMP EFK17 derived from LL-37 Terminal amidation and acetylation	➢ Increased conformational stability and proteolytic susceptibility—target organisms: *S. aureus* aureolysin and V8 protease; *P. aeruginosa* elastase	[64]
Amphiphilic peptides	Proteolytic degradation	Self-assembly of C_17_H_35_GR7RGDS peptide or Arginine nanoparticle	➢ Arginine imparts more positive charge and improves membrane interactions ➢ Augment the selectivity for healthy cells ➢ Strong activity against Gram-positive bacteria with minimal toxicity	[76]
Peptide (KIGAKI)_3_-NH_2_	Conformational stability	Stimuli-responsive hydrogel prepared by combining two AMP sequences with a central tetrapeptide linker	➢ Abrupt structural transformation from random coil to more stable β-hairpin conformation ➢ Form hydrogel in the presence of external stimuli like pH, heat, and ionic strength ➢ Inherent antibacterial activity against *E. coli* was preserved	[77]
AMP, SWLSKTAKKLFKKIPKKIPKKRFPRPR PWPRPNMI-NH 2, purity at >95%)	Less vascularization and prolonged inflammatory phase-Diabetic wound healing	Incorporation of hyaluronic acid-based hydrogels along with platelet-rich plasma (PRP)	➢ Suppresses inflammation ➢ Promotes angiogenesis and collagen deposition (incorporation of PRP) ➢ Effective against *S. aureus*, *E. coli*, and *P. aeruginosa* ➢ Fibroblast proliferation-improved wound healing in mice	[78]
Human antimicrobial peptide (AP-57)	Limited knowledge of its stability and efficacy	In situ gel formation using biodegradable poly (L-lactic acid)-Pluronic L35-poly (L-lactic acid) (PLLA-L35-PLLA) Thermosensitive biodegradable system	➢ High drug loading and encapsulation efficiency ➢ AP-57 showed release over an extended period ➢ Sol-to-gel conversion without any cross-linking agent once applied to wounds ➢ Reduced cytotoxicity and enhanced in vitro antioxidant activity ➢ Enhanced angiogenesis and increased collagen deposition-promoting cutaneous wound healing	[79]
Octapeptide (IKFQFHFD)	Potential pH-switchable antimicrobial effect	Effective acetylation and amidation at the N and C terminus of this peptide pH-responsive nanofiber-based hydrogels Incorporation of cypate (photothermal compound) and proline (pro-collagen compound)	➢ Antimicrobial activity at acidic pH (5.5–5.6), which is prevalent in chronic wounds ➢ At acidic pH, destabilization of nanofibers, releasing peptides ➢ Complete healing of MRSA-infected wounds in mice within 20 days	[80]
Hydrophilic peptide (dalargin)	Lower encapsulation efficiency in PLGA nanoparticles	Modifying the method of preparation Use of ionic additive SDS (sodium dodecyl sulphate)	➢ SDS improved the entrapment efficiency of dalargin with solvent diffusion (91.2%) and evaporation methods (68.6%)	[81]
Nisin	Electrostatic repulsion with divalent cations associated with bacterial cell surface	Liposomes of phosphatidylcholine (PC) and phosphatidyl glycerol (PG) were prepared	➢ PC/PG with ratio of 8:2 and 6:4 showed ~70–90% entrapment efficiency ➢ Peptides were stable within the liposomes at elevated temperatures and alkaline or acidic pH	[82]

**Table 3:** Improved peptide delivery systems for AMPs.

Peptide Delivery System	AMP	Description	Result	Reference
Mesoporous silica nanoparticles (MSNs)	Nisin A (bacteriocin isolated from *Lactococcus lactis* subsp. *Lactis*)	SBA-15 and MCM-41 type mesoporous nanomaterials prepared	➢ Limited proteolytic degradation ➢ MCM-41 type MSN provided the highest adsorption (pertaining to smaller pore size ~2.8 nm)	[83]
	Trichogin GA IV (short sequence), ampullosporin A (medium length sequence)	Continuous wave (CW) electron paramagnetic resonance (EPR) and pulsed electron-electron double resonance (PELDOR) techniques utilized for adsorption onto silica nanoparticles	➢ Conformational stability established for the AMPs	[84]
	Melittin	MSNs capped with β-cyclodextrin and magnetic core (adamantane) Melittin was loaded along with ofloxacin, and its release was compared with the free drug	➢ Higher suppression of *P. aeruginosa* biofilms ➢ Limited cell toxicity	[85]
Antimicrobial peptide conjugates	Aurein 2.2 (α-helical AMP)	Replace PEGylated conjugation with hyperbranched glycerol (HPG) conjugation Peptide density over conjugation measured as a function of antimicrobial activity	➢ Greater biocompatibility ➢ Non-toxic to fibroblasts ➢ Active against *S. aureus* and *S. epidermidis*	[86]
	Anoplin (decapeptide, short AMP)	Grafting over chitosan polymers Copper-catalyzed alkyne-azide coupling (CuAAC chemistry)	➢ Addresses the limitation of increased hemolytic activity associated with improving the antimicrobial potency of anoplin	[87]
	Nisin	Porous graphene oxide membrane used for conjugation	➢ 100% MRSA can be removed and destroyed using the developed membrane	[88]
Bacteria-absorbing sponge	Host defense peptides (HDPs) (peptidomimetics)	Guanidium-rich lipopeptide incorporated in liquid-crystalline hydrogel Trap and kill mechanism	➢ The developed sponge removes ~98.8% of bacteria ➢ Addresses the limitations of HDPs in topical formulations like prolonged preparation time, insignificant toxicity reduction, and inefficient bacterial capturing	[89]
Layered nanoclays	LL-37	Laponite-based nanoparticles	➢ Both bacterial flocculation and membrane lysis were observed upon LL-37 loading into laponite nanoparticles	[90]
Carbon nanotubes	TP359	Silver-coated carbon nanotubes were functionalized with TP359 in both covalent and non-covalent technique	➢ Addresses individual limitations of stability and toxicity in human cells for AMPs and carbon nanotubes, respectively ➢ Covalent functionalization gave synergistic antibacterial activity and reduced toxicity	[91]
Titanium nanoparticles	Lactoferrin-derived hLf1–11	Three different techniques of covalent immobilization were tested for antibacterial activity against oral strains (*Streptococcus sanguinis* and *Lactobacillus salivarius*) 3-Aminopropyltriethoxysilane (APTES) and polymer brush-based coatings with two different silanes	➢ ATRP (atom transfer radical polymerization) showed a greater decrease in bacterial attachment	[92]

## 8. Discussion and Outlook

AMR poses a significant challenge in the treatment of infections, as bacteria continue to evolve mechanisms to evade the effects of conventional antibiotics. AMPs, though promising, are not immune to resistance development. Enterococci, for example, have shown the ability to develop resistance to peptides, which can result in serious infections [34]. Delving into the ground molecular mechanisms responsible for the emerging resistance to AMPs is the major outlook in peptide antibiotics [93,94]. Susceptibility testing and other factors, similar to antibiotics, should be considered for the application of such AMPs to minimize the risk of bacterial resistance [95]. As mentioned earlier, several mechanisms of bacterial resistance against AMPs have already been defined; many of these, however, require major adaptations and further modifications concerning bacteria [96].

AMP delivery from the context of topical infections has always been shown to possess formidable challenges due to its large size and hydrophilicity. As a consequence, several systems like nanoparticles [47], cubosomes [66], nanostructured lipid carriers [67], mesoporous matrices [85,97], microgels [98], and hydrogels [99] have been explored and established. Despite these advancements, each system has its limitations of limited local residence time, limited bioadhesivity, fibroblast cell toxicity, and ROS toxicity. The enhancement of such existing topical delivery systems of AMP has been discussed in this paper both from the starting material (i.e., peptide improvement) (Table 2) and the formulation perspective (Table 3). In cases where the skin barrier is impaired, such as in atopic dermatitis, medical device approaches such as microneedles, sonophoresis, and iontophoresis have been explored to improve AMP delivery [100].

In conclusion, this review complements other literature on AMP delivery [6,16,17,18] by addressing specific gaps related to skin microbiome considerations, peptide stability, and market insights. It reinforces shared concerns about delivery system limitations and emphasizes optimization strategies as a path forward for AMP therapeutics.

From a future perspective, the targeting of genes responsible for emerging bacterial resistance against such peptides is currently being explored as a possibility for successful topical delivery of AMPs. For example, AMP sensing systems (a strategy to overcome the resistance mechanism, which is guided by the *Aps* gene regulated by the *dlt* operon in *S. aureus*) have been reported to confer sensitivity to certain AMPs [101].

Overall, careful consideration of all the factors, like conformational stability of peptides, microbiome nature (pH) at the site of infection, and proteolytic stability, shall lead to the development of an efficient AMP delivery system for topical infections.

## Data Availability

Not applicable.
